# Platelet specific knockout of integrin beta-3 (β3) reduces severity of necrotizing enterocolitis in murine neonates

**DOI:** 10.3389/fped.2025.1560242

**Published:** 2025-07-17

**Authors:** Marie Amalie Balamurugan, Balamurugan Ramatchandirin, Suneetha Desiraju, Arjun Subrramanya, Juanitaa George Raj, Megan M. Ferris, Zainab D. Lawal, Oluwabunmi O. Olaloye, Liza Konnikova, Krishnan MohanKumar

**Affiliations:** ^1^ Department of Biochemistry and Molecular Biology, University of Nebraska Medical Center, Omaha, NE, United States; ^2^ Department of Pediatrics, Johns Hopkins University School of Medicine, Baltimore, MD, United States; ^3^ Child Health Research Institute, Children’s Nebraska and University of Nebraska Medical Center, Omaha, NE, United States; ^4^ Department of Pediatrics, Yale University School of Medicine, New Haven, CT, United States; ^5^ Department of OB-GYN and Reproductive Sciences, Yale University School of Medicine, New Haven, CT, United States; ^6^ Program in Human Translational Immunology, Yale University School of Medicine, New Haven, CT, United States; ^7^ Program in Translational Biomedicine, Yale University School of Medicine, New Haven, CT, United States; ^8^ Department of Immunobiology, Yale University School of Medicine, New Haven, CT, United States; ^9^ Center for Systems Engineering and Immunology, Yale University School of Medicine, New Haven, CT, United States; ^10^ Department of Pediatrics, University of Nebraska Medical Center, Omaha, NE, United States

**Keywords:** necrotizing enterocolitis, integrin beta-3, platelet, monocyte, platelet-monocyte aggregation

## Abstract

**Introduction:**

Necrotizing Enterocolitis (NEC) is the most impactful gastrointestinal disease of premature neonates and preclinical evidence shows that the event of platelet activation is an important pathophysiological contributor during NEC-like injury in murine neonates. Integrin αIIb/β3 (glycoprotein [GP]IIb/IIIa) is the primary platelet activation marker showing increased platelet-monocytes aggregation during NEC-like injury. The present study investigates whether platelet lineage-specific deletion of integrin-β3 reduces NEC-like injury in murine neonates.

**Methods:**

C57BL/6 and integrin-β3^−/−^ mouse pups were subjected to trinitrobenzene sulfonic acid (TNBS)-induced NEC-like injury (*n* = 6/each group). Monocyte-platelet aggregation was measured by flow cytometry and immunofluorescence. Plasma levels of intestinal injury markers (FABP2, CRP, CXCL2 and SAA) and inflammatory cytokines (TNF-α, IL-1β, IL-6 and IL-1α) were measured by ELISA and multiplex array respectively. Intestinal inflammatory responses were confirmed by qRT-PCR.

**Results:**

Integrin-β3-associated platelet-monocyte aggregation was significantly observed in the intestine and blood of murine NEC-like injury and in the human NEC intestine. Platelet-specific deletion of integrin-β3's exon-1 leads to inhibition of platelet-monocyte aggregation in circulating blood and intestine, thus reducing the resulting intestinal injury and the level of inflammatory activation cytokines in the blood.

**Conclusion:**

Monocyte-platelet aggregation is an important pathophysiological event and the blockade of integrin-β3 merits a potential therapeutic target in NEC.

## 1 Introduction

Necrotizing Enterocolitis (NEC) is the most impactful gastrointestinal disease of premature neonates, affecting 5%–15% of infants with a birth weight <1,500 g (1–4). NEC is a leading cause of death in infants born at 22–28 weeks gestation (5). Nearly 75%–90% of infants with confirmed NEC develop thrombocytopenia (<150 × 10^9^/L) within a few hours of disease onset and the degree and duration of thrombocytopenia correlate with the severity of bowel injury (6–16). In mice, NEC-like injury is associated with thrombocytopenia that occurs despite increased megakaryopoiesis, indicating that platelet consumption is the likely kinetic mechanism of thrombocytopenia, rather than decreased production (11, 12). Platelets contain large amounts of inflammatory mediators such as platelet activation factor (PAF), various chemokines, reactive oxygen species, β-thromboglobulin, platelet-derived growth factor, thromboxane, hydroxyeicosatetraenoic acid (5-HETE), 5HT, and transforming growth factor-β, to name a few (17–22). Activated platelets release these and other inflammatory and vasoconstrictive factors, and therefore, constitute a plausible cellular mechanism for bowel injury and the development of histopathological changes pathognomonic of NEC (inflammation and coagulation necrosis, indicative of microvascular ischemia) (23).

Integrin-β3 (Itgb3) is a cell-surface receptor on platelets and also a subunit that can bind with Itga2b (αIIb) to form integrin αIIbβ3 (also known as glycoprotein IIb/IIIa or CD61), which is the most abundant integrin on the platelet surface (24), and this higher expression projects platelet activation within 3 h after TNBS (trinitrobenzene sulfonic acid)-induction for murine NEC-like injury (12, 16). The αIIbβ3 complex protects glycoproteins from proteolytic digestion; thus, if either integrin αIIb or integrin-β3 is absent or defective, the other subunit will be rapidly degraded (25–27). Based on these studies, we posited that platelet-specific depletion of integrin-β3 inhibits the formation of αIIbβ3 complex thus reducing the platelet-activation-associated inflammatory response.

We showed recently that platelet activation is the central pathophysiological NEC event that causes platelet depletion, as well as platelet hyperaggregability due to dense granule discharge that augments mucosal damage and the associated systemic inflammatory response (11, 12, 16). We have also reported that NEC is marked by the recruitment of monocyte/macrophage-rich leukocyte infiltrates that display signs of inflammatory activation and produce inflammatory cytokines (28–31). Platelets can attach to and activate leukocytes (22) and platelet–monocyte aggregates have been associated with various diseases, especially in neonatal sepsis (32). However, our understanding of their role in NEC is limited. Given the crucial role of platelet activation and its hyperaggregability in NEC, we now tested whether activated platelets may aggregate with monocytes through activation marker integrin β3-involved αIIbβ3 complex and whether targeting by silencing platelet-specific integrin β3 expression thus inhibits the formation of αIIbβ3 complex and attenuates NEC-like injury and systemic inflammatory response.

## 2 Methods

### 2.1 Animals

Animal studies were approved by the Institutional Animal Care and Use Committee at Johns Hopkins University and University of Nebraska Medical Center. To generate mouse pups with the deletion of integrin β3 in the platelet lineage, the mice with floxed alleles (C57BL/6-*Itgb3^tm1.1Wlbcr^*/Jax) were crossed with mice carrying *Cre* recombinase under the control of the platelet factor-4 (PF4) (C57BL/6-Tg(Pf4-icre)Q3Rsko/Jax) promoter. The sample size was estimated for intestinal inflammation at α = 0.05% and 80% power (Lehmann's method for non-Gaussian data). Pups were housed with and nursed by the dam throughout the study period. As described previously (12, 29), TNBS (trinitrobenzene sulfonic acid)-enterocolitis was induced in 10-day-old C57BL/6 and transgenic mice of PF4-*Cre^+^* and integrin-β3 mice by administering trinitrobenzene sulfonic acid (TNBS, catalog #92822, Sigma; 2 doses of 50 mg/kg in 30% ethanol, wt/vol) by gavage and rectal instillation, respectively. C57BL/6 controls and PF4-*Cre* controls received vehicle alone (30% ethanol) by gavage and rectal instillation. Pups from each litter were randomly assigned to these two study groups. Animals were monitored every 3 h and were euthanized if they developed physical distress or at 24 h using carbon dioxide inhalation followed by cervical dislocation. Blood was collected at 0, 3, 9 and 24 h, intestine and spleen tissue were harvested at 24 h after induction of NEC-like injury for further analysis. Histopathological grading of intestinal injury has been previously described (12, 29, 33).

### 2.2 Human intestinal tissue samples

De-identified, archived paraffin-embedded tissue specimens of human NEC and uninflamed human premature intestine resected for indications other than NEC were obtained from existing biorepositories at Yale University with approval of the institutional board (IRB# 2000028415).

### 2.3 Immunofluorescence

We used our previously described protocols (12, 33). Formalin-fixed paraffin-embedded tissues were deparaffinized and processed for antigen retrieval (EZ-AR Common solution, Biogenex, San Remon, CA, catalog #HK545-XOK), digested with Proteinase K (20 µg/ml, 10 min; Promega, Madison, WI; catalog #EO0491), and blocked for 30 min (SuperBlock T20 blocking buffer, ThermoFisher, Waltham, MA; catalog #37516). Tissue sections then were incubated overnight at 4°C with rabbit anti-mouse Ly6C (dilution 1:200; Abcam catalog #ab77766) and rat anti-mouse/human CD41 (dilution 1:200; ThermoFisher catalog #MA5-16875). Human intestine tissue sections were stained with mouse anti-CD14 (dilution 1:100; Abcam, catalog #ab182032) and rat anti-mouse/human CD41 (dilution 1:200; ThermoFisher catalog #MA5-16875). Antibodies were selected after reviewing the validation information provided on the manufacturers' websites. Secondary staining was performed with Alexa 488, Alexa 546 or Alexa 633-conjugated antibodies × 60 min (Invitrogen, San Diego, CA). Nuclear staining was obtained with 4',6-diamidino-2-phenylindole (DAPI; Prolong Gold Antifade Mountant, ThermoFisher; catalog #P36930). Imaging was performed in Nikon C2 Confocal microscopy equipped with Nikon LUN4 and analyzed using Nikon NIS elements software.

### 2.4 Flow cytometry

Blood was collected from anterior facial vein puncture or at sacrifice using a 25G needle on a syringe containing 1 vol ACD buffer (acid-citrate-dextrose) for 6 vol blood. Red blood cells were immediately lysed by incubating with 1X RBC lysis buffer (ThermoFisher, catalog #00-4333-57) for 10 min at room temperature, then washed with phosphate buffered saline (PBS; ThermoFisher, catalog #J67670-K2) and centrifuged at 300 × g (10 min) to obtain cell pellets. Cells were resuspended in Cells Staining Buffer (Biolegend; catalog #420201). Intestinal single-cell suspensions were prepared as per previously described (33). Briefly, intestinal tissue was washed with Hank's balanced salt solution (ThermoFisher, catalog #88284) containing 1 mM dithiothreitol (Millipore Sigma, catalog #646563) to remove mucus, and then treated with HBSS containing 1 mM EDTA (Millipore Sigma, catalog #20-158) for 20 min at 37°C. Next, it was treated with HBSS containing 1 mM collagenase type IV (Millipore Sigma, catalog #C4-BIOC) for 2 h at 37°C. The digested intestinal suspensions passed through 70 µm filters and were centrifuged at 400×g for 10 min. Cell pellets were then resuspended in Cell Staining Buffer (Biolegend; catalog #420201). Blood, spleen, and intestinal derived cells were stained with the Hoechst 33342 ready flow reagent (ThermoFisher, catalog #R37165) for DNA and antibodies with CD11b (dilution 1:25; clone #M1/70), Ly6C (dilution 1:25; clone #HK1.4), CD41 (dilution 1:25; clone #MWReg30) and CD61/integrin β3 (dilution 1:20; clone 2C9.G2). Data was acquired on a BD LSR-II flow cytometer and analyzed using the software package FlowJo version 10.5.3 (Becton Dickinson, Franklin Lakes, NJ) with our existing flow gating strategies (33). Some of the stained cells were acquired on an AMNIS Flowsight Imaging Flow Cytometer with bright field and multiple fluorescent images of each cell, image analyzed with IDEAS software.

### 2.5 ELISA and Multiplex assay

Commercially available enzyme immunoassays were used to measure murine FABP2, CXCL2, CRP, and SAA (MyBioSource, San Diego, CA; catalog #MBS1751561, MBS824972, MBS264470 and MBS2500351) in plasma of experimental transgenic animal groups based on the manufacturer's protocol. The level of major inflammatory cytokines Tumor Necrosis Factor-alpha (TNF-α), Interleukin-1 beta (IL-1β), Interleukin-6 (IL-6) and Interleukin-1 alpha (IL-1α) were quantified in the plasma by using the Magnetic Bead Kit (Millipore Sigma). Samples were run in the MAGPIX instrument (Luminex Corp.) and the results were analyzed using xPONENT software (Luminex Corp.).

### 2.6 qPCR

We used a standard reverse transcriptase-polymerase chain reaction (RT-PCR) to measure mRNA expression of the inflammatory cytokines (TNF-α, IL-1β, IL-6 and IL-1α) in the intestine (29). Primers were designed using the Beacon Design software (Bio-Rad, Hercules, CA). Data from transgenic mice experiments were compared against the 18S rRNA by the 2–ΔΔCT method and normalized with Wild Type controls.

### 2.7 Statistical analyses

Statistical analysis was performed using GraphPad Prism software, version 9.4.1 (GraphPad Software, La Jolla, CA). Differences were considered significant at *P* < 0.05.

## 3 Results

### 3.1 Platelet-monocyte aggregates in human NEC

**Figure 1:**
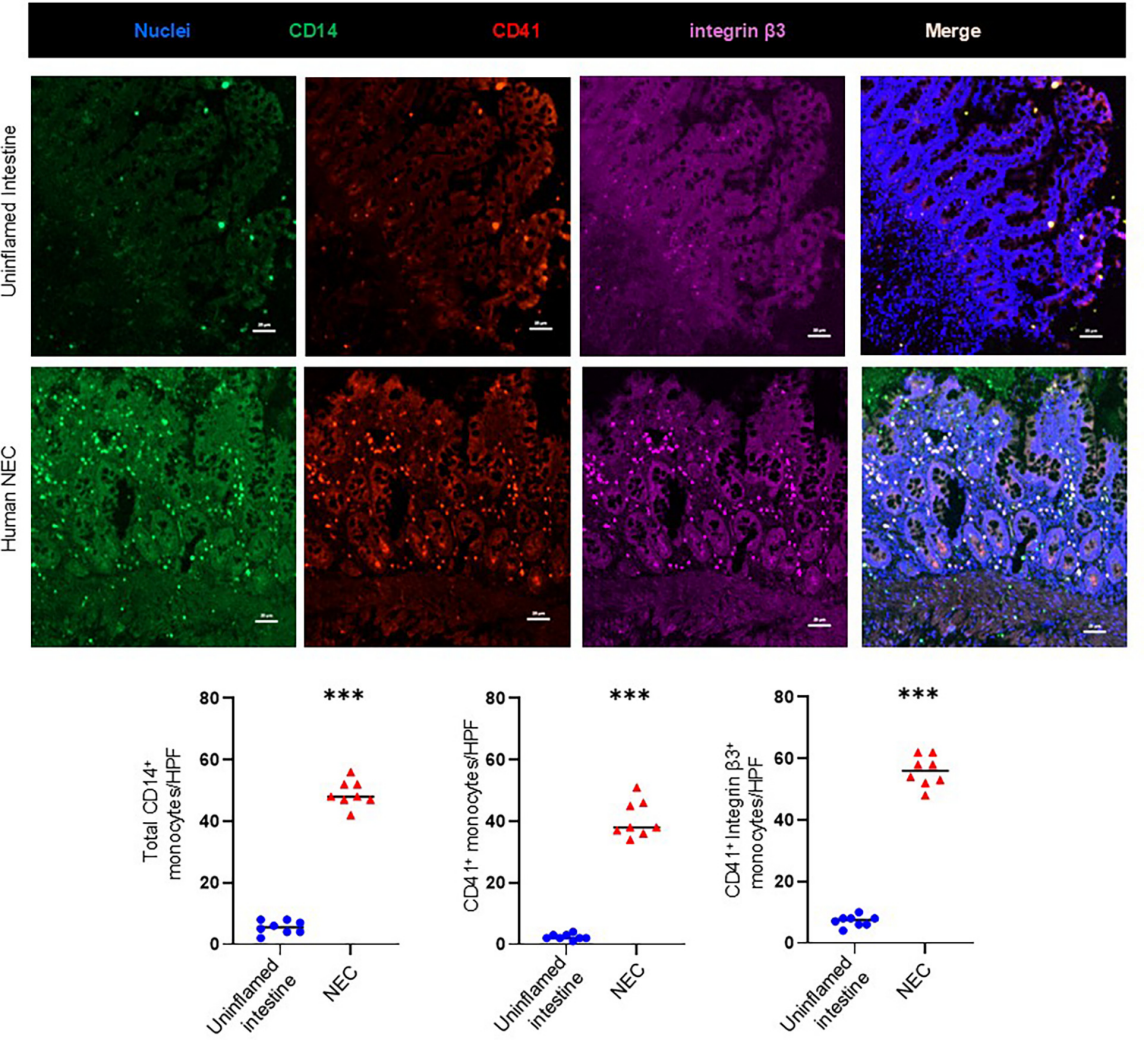
Platelet-monocyte aggregates in human NEC. Representative fluorescence photomicrographs (200×) of the uninflamed premature intestine (Top) and inflamed mucosa of NEC lesions (Bottom) show immunoreactivity for CD41 (red) in intestinal recruited monocytes (CD14^+^, green) with integrin β3 (purple). CD41 immunoreactivity was more extensively co-localized in monocytes with integrin β3 (Scale bar, 20 μm) in NEC inflamed intestine. Scatter plots below summarize the total monocytes (CD14^+^)’ fluorescence intensity with the CD41^+^ platelets and integrin β3 in control and NEC intestine, respectively. *N* = 8 patient samples/group; Mann–Whitney *U*-test; ***P < 0.001.

To localize platelet-monocyte aggregates in Necrotizing Enterocolitis (NEC) lesions, we first immunostained the tissue samples of uninflamed premature intestine (that had been surgically resected for conditions other than NEC) as well as human NEC samples for the platelet antigen CD41/GPIIb, CD14, and integrin-β3. As shown in representative photomicrographs and summarized in the bar diagram (Figure 1), human NEC was characterized by a significantly higher density of inflammatory monocyte infiltrate (total CD14^+^ monocyte) than control intestinal tissue (5.5 ± 0.5 cells/HPF in control tissue vs. 42.5 ± 0.4 cells/HPF in NEC; *P* < 0.001). Tissue resected for human NEC showed stronger CD41 immunoreactivity, which was localized on CD14^+^ monocytes, and not in microthrombi in the gut microvasculature (2.0 ± 0.4 cells/HPF in control tissue vs. 36 ± 0.9 cells/HPF in NEC; *P* < 0.001). We (29–31, 33, 34) and others (35, 36) have previously described that NEC was associated with a prominent inflammatory monocyte/macrophage infiltrate. Because monocytes do not synthesize CD41, any CD41 staining on monocytes is believed to be due to adherent platelets (37). Moreover, we found a strong co-localization of CD14^+^ monocyte with integrin-β3 expressing CD41^+^ platelets indicating the presence of monocyte-platelet interactions through integrin-β3.

### 3.2 Platelet-monocyte aggregates in murine NEC-like injury

**Figure 2:**
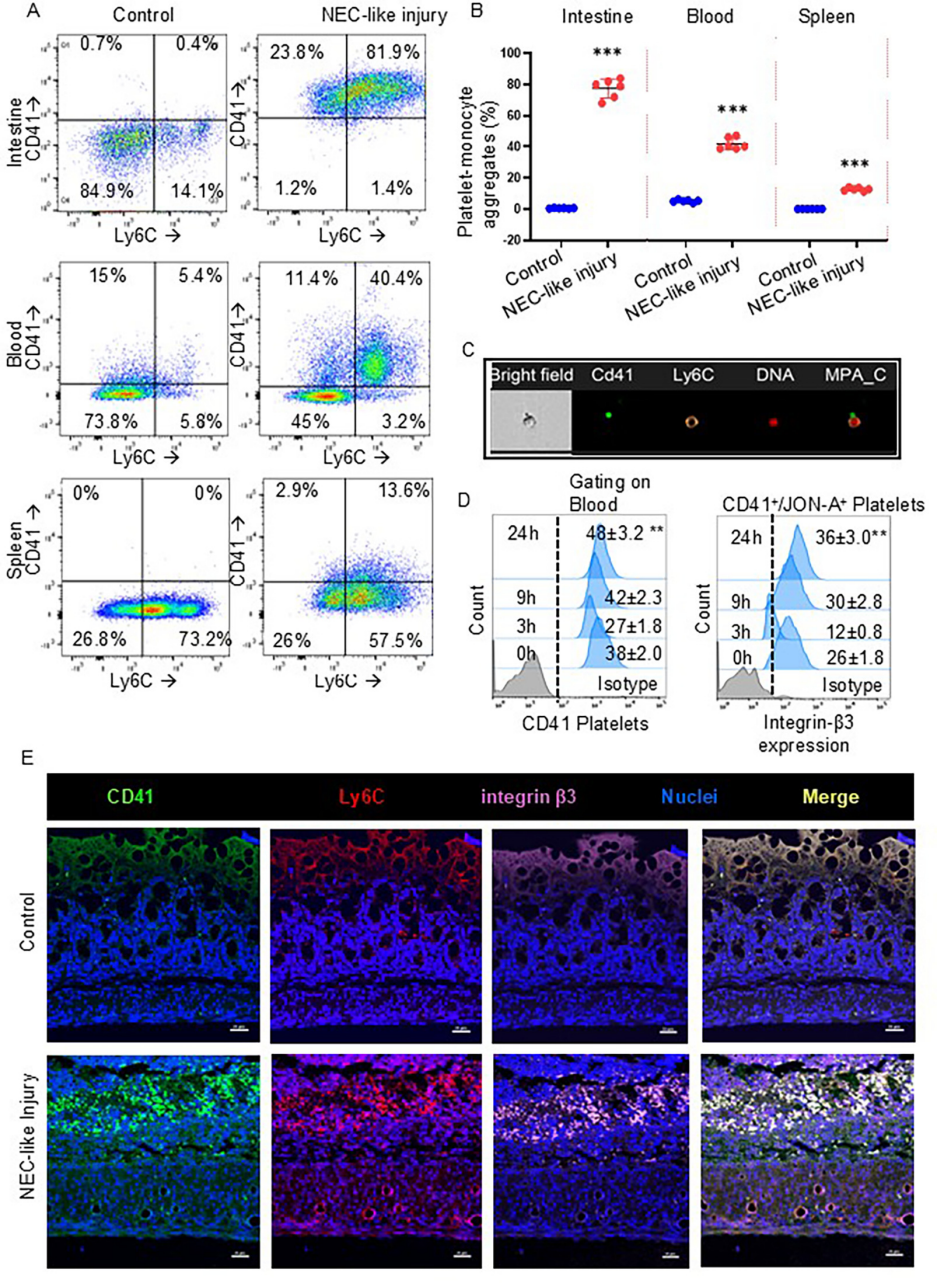
Platelet-monocyte aggregates in murine NEC-like injury. **(A)** Representative scatter plots and gating strategy from the intestine, blood, and spleen cells of control and NEC-like injury show the platelet-monocyte aggregation by the presence of both CD41 ^+^ Ly6C^+^ fractions which were enriched in NEC-like injury. **(B)** Scatter plots (Right) summarize the percentage of CD41 (+) and Ly6C (+) cells in each group. Šídák's multiple comparisons tests, ***P < 0.001 vs. respective controls. Data represents 6 mice per group. **(C)** Representative image of circulating platelet-Ly6C*^+^* monocyte cell aggregates that are seen in NEC-like injury as obtained from imaging flow cytometry. Platelets are recognized by anti-CD41 (green) while Ly6C is recognized by anti-monocyte (orange) and nuclei DNA (red) with bright fields. **(D)** Fluorescence-activated cell-sorting histograms show platelet expression of integrin-β3 at different time points after NEC-like intestinal injury in the blood. Controls did not change over time and have not been depicted; *N* = 6 mice/group, Kruskal–Wallis H test with Dunn's posttest. **P < 0.01 vs. control (0 h). **(E)** Fluorescence photomicrographs (ileocecal; magnification ×800) showed the presence of numerous Ly6C monocytes (red) with CD41^+^ platelets (green) and with integrin-β3 (purple) in NEC-like injury than control. Scale bar = 25 µm.

To investigate the hypothesis that platelet activation leads to hyperaggregability with monocytes and triggers a characteristic inflammatory response in murine NEC-like injury, we have used our murine model of intestinal injury from TNBS (trinitrobenzene sulfonic acid) by gavage and enema in 10-day-old mouse pups and observed these animals for up to 24 h. We first examined cell suspensions from enzymatically digested intestinal tissue samples, blood, and spleen by flow cytometry. Similarly to human NEC, the murine CD41^+^ platelets have strong immunoreactivity localized with Ly6C^+^ monocytes in the intestine (0.56 ± 0.06% of cells in control tissue vs. 77.5 ± 2.52% of cells in murine NEC-like injury; *P* < 0.001) and blood (5.16 ± 0.33% of cells in control blood vs. 42.01% ± 1.53% of cells in blood of murine NEC-like injury; *P* < 0.001); and minimal reactivity in spleen (0.08% ± 0.04% of cells in control tissue vs. 12.6% ± 0.42% of cells in murine NEC-like injury; *P* < 0.001) (Figures 2A,B). Accordingly, the Amnis imaging flow cytometer detected the aggregation of CD41^+^ platelets with Ly6C^+^ monocytes in blood during TNBS-induced NEC-like injury (Figure 2C). Regarding the time kinetics of platelet activation during NEC-like injury, we investigated blood samples collected at various time points (0, 3, 9 and 24 h) after TNBS induction using flow cytometry analysis, which showed that activation occurs between 3 and 9 h with higher expression of integrin-β3 (identified by using clone JON-A antibody) on CD41^+^ platelets and steadily increased until 24 h (Figure 2D). These findings were also confirmed by immunofluorescence imaging, which showed newly recruited Ly6C^+^ monocytes co-localized strong immunoreactivity with CD41^+^ along with integrin-β3 (Figure 2E).

### 3.3 Platelet-specific knockdown of integrin-β3 prevents platelet-monocyte aggregates in murine NEC-like injury

**Figure 3:**
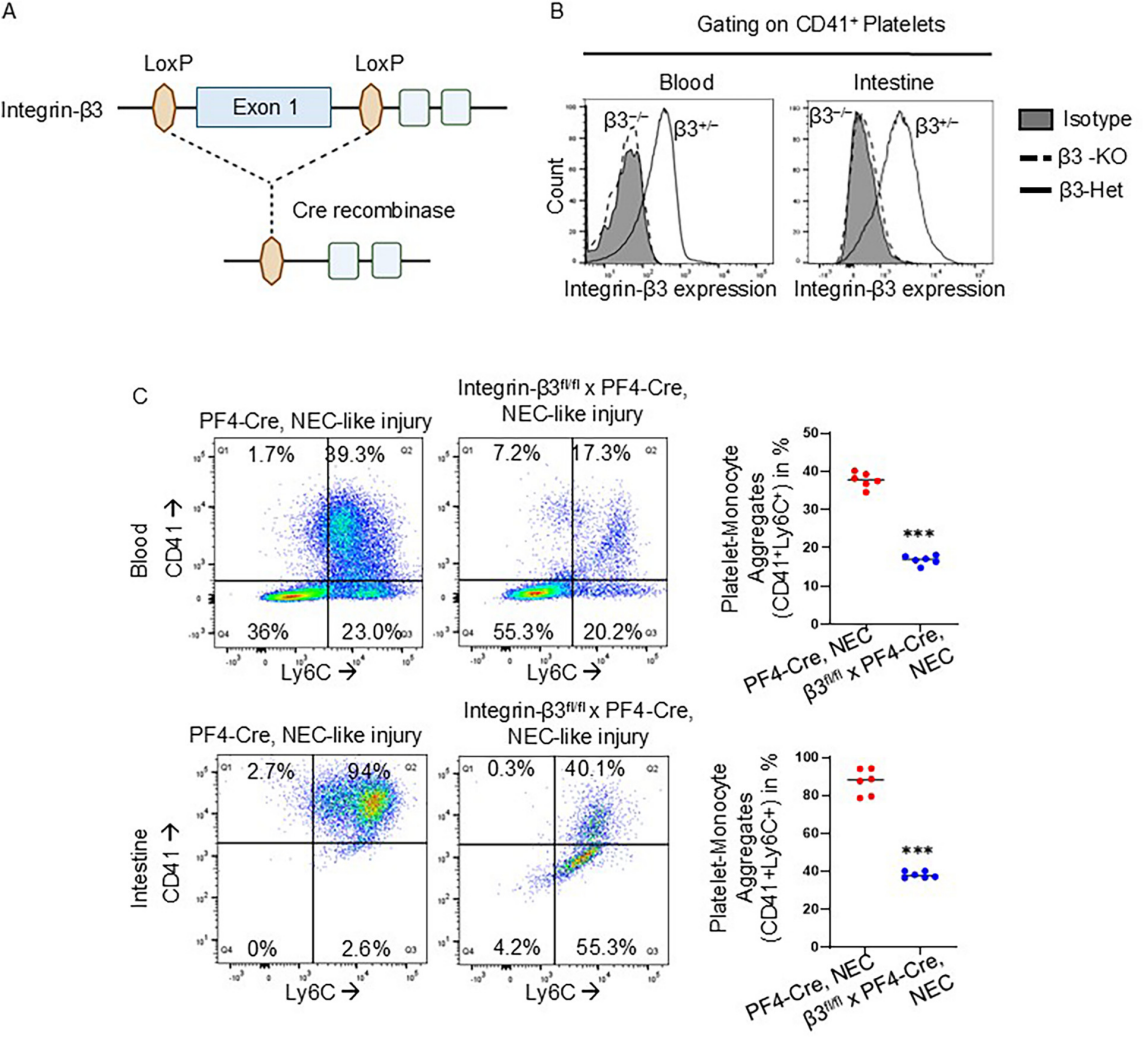
Platelet-specific knockdown of integrin-β3 prevents platelet-monocyte aggregates in murine NEC-like injury. **(A)** Schematic of gene targeting strategy. Exon 1 of the integrin-β3 gene, flanked by LoxP sites, was excised by PF4 promoter-driven expression of Cre recombinase. **(B)** FACS analysis demonstrating that integrin-β3 deletion was specific to the platelets of integrin-β3^fl/fl^ PF4-*Cre*^+^ (integrin β3^−/−^) mice (*continuous line*) in both blood and intestine during NEC-like injury, no deletion occurred (*dotted line)* in integrin-β3^fl/wt^PF4-*Cre*+ (integrin-β3^+/−^). **(C)** Representative flow cytometry scatter plots with gating strategy and scatter plots from blood and intestine cells of PF4-*Cre*^+^ and integrin-β3^fl/fl^ PF4-*Cre*+ (integrin β3^−/−^) mice show the reduced platelet-monocyte aggregation in NEC-like injury.

To ascertain the role of platelet-monocyte aggregates in murine NEC-like injury, we used platelet factor-4 (PF4)-*Cre*-recombinase-mediated excision to delete the exon 1 on integrin-β3 gene specifically in the platelet lineage (Figure 3A). To confirm platelet-specific gene deletion, we used flow cytometry analysis and found successful deletion of integrin-β3 on CD41^+^ platelets in both blood and intestinal cells of TNBS-induced NEC-like injury in integrin β3^fl/fl^ PF4-*Cre* + (integrin-β3^−/−^) murine pups than integrin β3^fl/wt^PF4-*Cre* + (integrin β3^+/−^) (Figure 3B). Based on emerging information that platelet activation leads to expression of integrin-β3 on their surface and leads to formation of monocyte-platelet aggregates, we next performed flow cytometry analysis for blood and intestine cells from TNBS-induced PF4-*Cre*^+^ (control) and integrin-β3^−/−^ pups. A greater percentage of platelet-monocyte aggregation (CD41^+^ Ly6C^+^) was observed in blood (0.62 ± 0.02% of cells in NEC-like injury controls vs. 0.11 ± 0.005% of cells in NEC-like injury induced integrin-β3^−/−^; *P* < 0.001) and intestine cells (3.00 ± 0.03% of cells in NEC-like injury controls vs. 0.20 ± 0.004% of cells in NEC-like injury induced integrin-β3^−/−^; *P* < 0.001) of control PF4-*Cre*^+^ mice after TNBS-induced NEC-like injury, whereas integrin β3^−/−^ murine pups showed significantly reduced platelet-monocyte aggregates after TNBS-induction (Figure 3C). Based on these data, we postulated that integrin-β3 is able to heterodimerize with αIIb (CD41) and leads to platelet activation which then causes platelet-monocyte aggregation, and therefore, we next sought to define the role of platelet-monocyte aggregation in NEC-like injury.

### 3.4 Inhibition of platelet-monocyte aggregations reduces NEC-like intestinal injury in murine neonates

**Figure 4:**
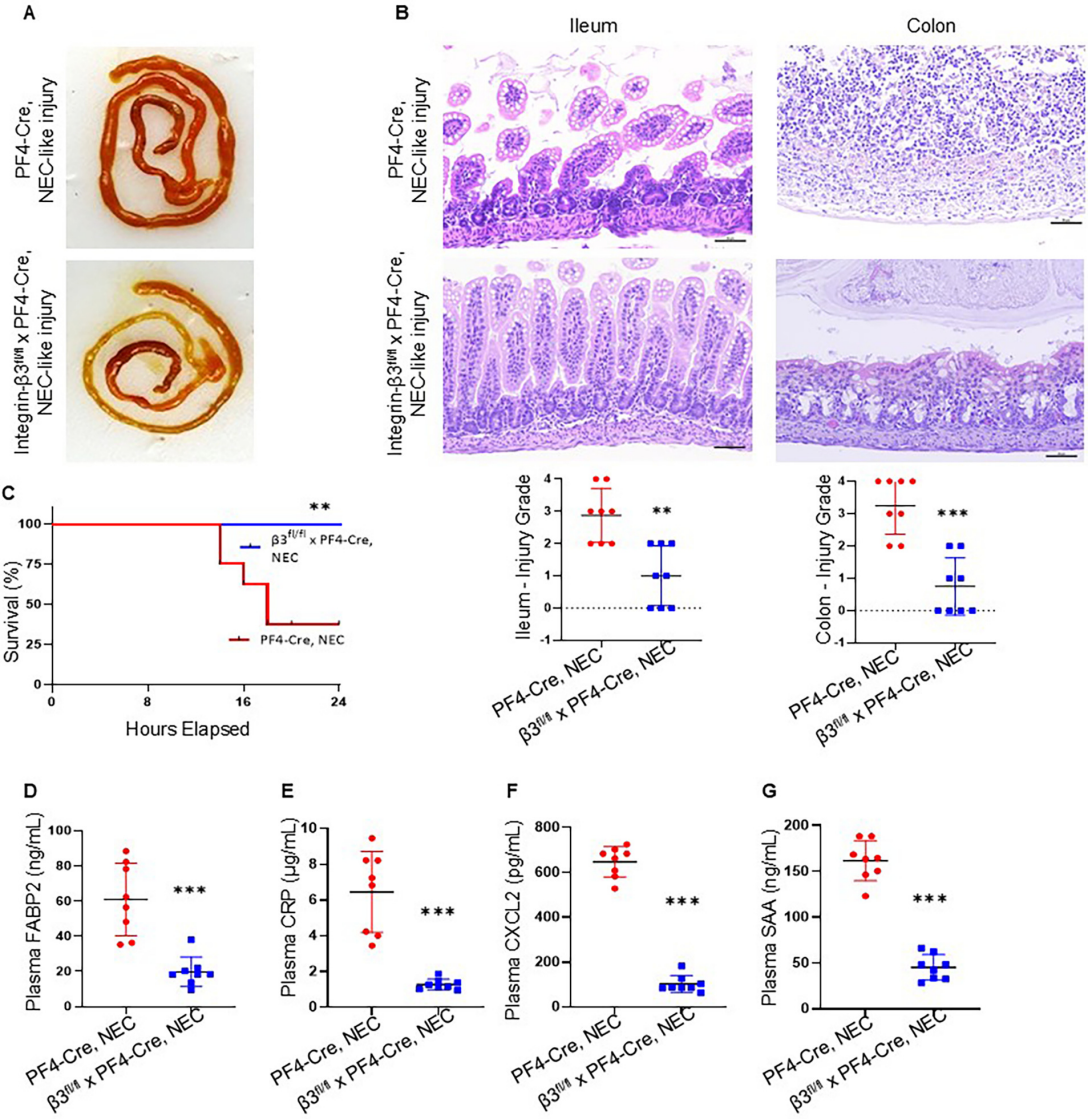
Inhibition of platelet-monocyte aggregates reduces the NEC-like intestinal injury in murine neonates. (A) Representative photographs show ileal and colonic injury in PF4-*Cre*+, but not in the integrin-β3^fl/fl^ PF4-*Cre*+ (integrin β3^−/−^) mice during NEC-like injury. **(B)** (*top*)*.* Hematoxylin and eosin (H&E)-stained photomicrographs of the ileum and colon from PF4-*Cre*+ show increased inflammatory cellularity in the lamina propria, but not in the integrin-β3^fl/fl^ PF4-*Cre*+ (integrin β3^−/−^) mice after NEC-like injury. Scale bars = 25 µm. (*bottom*) Scatter plots summarize ileocecal injury in grade. *N* = 8 animals per group of PF4-*Cre*+ and integrin-β3^fl/fl^ PF4-*Cre*+ (integrin β3^−/−^) with NEC-like injury. **(C)** Kaplan–Meier curves below show survival with NEC-like intestinal injury of integrin-β3^fl/fl^ PF4-*Cre*+ (integrin β3^−/−^) and PF4-*Cre* mice; *N* = 8 mice each group, Mantel-Cox log-rank test, **P < 0.01. **(D–G)** Plasma FABP2, CRP, CXCL2, and SAA in the two groups of PF4-*Cre*+ and integrin-β3^fl/fl^ PF4-*Cre*+ (integrin β3^−/−^) mice after TNBS induced NEC-like injury. *N* = 8 mice/group; Mann–Whitney U test, ***P < 0.001 vs. PF4-*Cre*+ intestinal injury.

We have described earlier that TNBS-induced NEC-like injury cause more severe injury marked by coagulative necrosis, inflammation, submucosal edema/separation and interstitial hemorrhages in C57BL6 murine pups (12, 29). A similar pattern of injury was noted in PF4-*Cre*^+^ mice during NEC-like injury. Interestingly, integrin-β3^−/−^ mouse pups showed improved survival and reduced severity of NEC-like injury without an increase in hemorrhages in the injured intestine (Figures 4A–C). Consistent with these histopathological findings, there was additional evidence for reduced epithelial injury evidenced by lower plasma FABP2 (Figure 4D). Attenuation of the systemic inflammatory response was also observed with lower plasma levels of CRP, CXCL2, and SAA (Figures 4E–G).

### 3.5 Murine NEC-like injury-associated inflammatory response is reduced in platelet-specific integrin-β3 knockdown mouse pups

**Figure 5:**
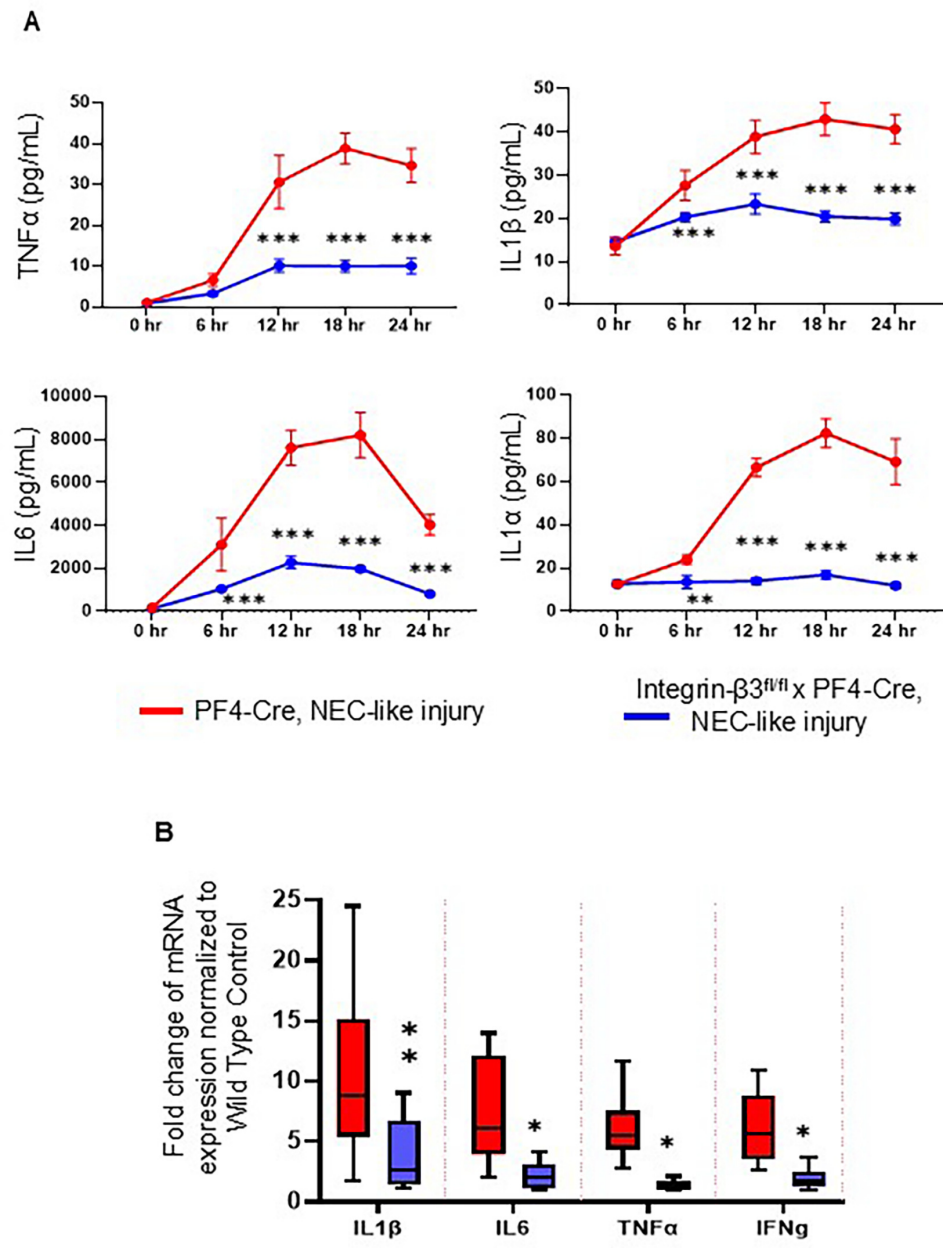
Murine NEC-like injury-associated inflammatory response is reduced in platelet-specific integrin-β3 knockdown mouse pups. **(A)** *Scatter* plots show plasma TNF-α, IL-1β, IL-6 and IL-1α) levels at different time intervals after TNBS-induction in PF4-*Cre*^+^ (red line) and integrin-β3^fl/fl^ PF4-*Cre*+ (integrin β3^−/−^) mice (blue line) and measured by the Milliplex cytokine array. *N* = 8 mice per group. Šídák's multiple comparisons tests, ***P < 0.001 vs. respective timed PF4-*cre* control with NEC-like injury. (B) Scatter bar diagrams summarize inflammatory cytokine expression in the PF4-*Cre*^+^ and integrin-β3^fl/fl^ PF4-*Cre*+ (integrin β3^−/−^) mice, measured by reverse transcriptase-quantitative polymerase chain reaction (RT-qPCR). *N* = 8 mice per group; Šídák's multiple comparisons tests, *P < 0.05, **P < 0.01.

We next sought to evaluate the inflammatory responses in integrin-β3^−/−^ mouse pups by Milliplex cytokine array in plasma and qPCR assay for inflammatory cytokines in intestinal tissue after NEC-like injury. Existing data suggest that platelet-monocyte aggregates may show increased inflammatory response in the blood circulation of various diseases (24). Indeed, TNBS-induction acutely increased the inflammatory cytokines (IL-1β, IL-6 and IL-1α) within 3 h (TNF-α increased at 6 h) post TNBS administration in PF4-*Cre*^+^ murine pups, which might have been due to stronger platelet-monocyte aggregation in these mice. Notably, integrin-β3^−/−^ murine pups showed no/minimal changes within 3 h and inflammatory cytokine levels were unchanged until sacrifice at 24 h, indicating that platelet-monocyte aggregation is the major contributor to NEC-like injury-associated inflammatory response (Figure 5A). In addition, integrin-β3^−/−^ mouse pups showed significant reduction of key inflammatory genes in intestinal tissues compared to PF4-*Cre*^+^ mice (Figure 5B) indicating that inhibition of platelet-monocyte aggregates reduces the severity of mucosal inflammation, which is the key event occurring during the incidence of NEC.

## 4 Discussion

We present a detailed preclinical investigation of integrin β3 and its imperative role in platelet-monocyte aggregation during NEC, with evidence of its importance demonstrated by the paucity of inflammation in its absence. This study confirms the findings of prior work showing that the interaction of platelets with monocytes has physiological inflammatory consequences and contributes to the increasing body of evidence indicating that they participate in the propagation of inflammatory damage.

We detected a marked increase in the number of monocytes in our tissue samples of human NEC and nearly all monocytes co-localized with CD41 staining. We have previously reported that leukocyte infiltration consists primarily of monocyte/macrophage infiltration, and that activation of these infiltrates is a prominent pathophysiological feature in human pathological specimens of NEC (29–31). The detection of CD41 and integrin-β3 staining on monocytes thereby provides evidence for platelet-monocyte aggregation during NEC as CD41 is not naturally expressed on monocytes (37). Existing studies demonstrate that the primary pathophysiology of NEC is transmural inflammation of the intestine leading to intestinal perforation as well as bacterial translocation, with ensuing systemic inflammation and/or sepsis. The clinical (38) and pre-clinical (39) observation in sepsis have shown circulating platelet-monocyte aggregates significantly elevated with 20-fold elevated mean fluorescence intensity of CD41 on monocytes. Our findings support the phenomenon of inflammation-related platelet-monocyte aggregation in blood and this study shows the same aggregation in the intestinal tissue section of NEC.

We recently described (12) that platelet activation was observed with higher expression of αIIb/β3 (glycoprotein [GP] IIb/IIIa), CD31/platelet endothelial cell adhesion molecule (PECAM)-1, and P-selectin (CD62P) at 3 h after TNBS administration, and that platelet activation was an early event during murine NEC-like injury. Activated platelets release a variety of inflammatory and vasoconstrictive factors, and therefore, constitute a plausible cellular mechanism for bowel injury and the development of histopathological changes pathognomonic of NEC (coagulation necrosis, which indicates microvascular ischemia, and inflammation) (23). In this study, we detected platelet-monocyte aggregation predominantly in the intestine of NEC-like injury that might be due to acute expression of integrin-β3 only on activated platelets. Integrins α2b and β3 constitute the glycoprotein GPIIb/IIIa, which is the common mediator in inside-out signaling cascades activated by thrombin, ADP, and Txa2 (39). Interestingly, the median fluorescence intensity of surface expression of integrin-β3 rose nearly 2× within 3–9 h in NEC-like injury platelets compared to resting platelets from P10 pups. As we reported (12) earlier increased expression of the high-affinity conformation of GPIIb/IIIa (binds JON/A antibody) at 3 h after initiating the NEC protocol, our findings indicate that neonatal platelets can rapidly mobilize intracellular GPIIb/IIIa to the surface during NEC. Our findings with intestinal tissue immunostaining of monocyte, platelet, and its activation marker integrin-β3 during NEC-like injury resembles the human NEC pathophysiology in the context of platelet-monocyte aggregation.

We report successful specific deletion of integrin-β3 on platelets using PF4-*cre* mice to prevent monocyte-platelet aggregation thus ameliorating NEC-like injury. PF4-cre transgenic mice have been widely used for the generation of lineage-restricted gene knockouts for studying megakaryocytes and platelets (40). Integrin-β3 is expressed on platelets and megakaryocytes in association with integrin αIIb (CD41) or integrin αV (CD51) on endothelial cells, monocytes and osteoclasts (41). Monocytes may express low levels of integrin-β3 on their surface and component of the vitronectin receptor (VNR) complex which mediates the binding of platelets to immobilized vitronectin without prior activation (42). PF4 (CXCL4) is expressed in monocytes and macrophages, in this case, either PF-*cre*-based deletion of integrin-β3 on platelets or monocytes might facilitate the inhibition of either αIIb/IIIa complex or αIIb/αV complex respectively leading to inhibition of platelet activation followed by aggregation.

The finding that inhibition of platelet-monocyte aggregation reduces intestinal injury during NEC is intriguing. These findings are consistent with existing information that blocking the platelet-leukocyte (monocyte or neutrophil) aggregation either P-selectin (CD62P) (43) and CD40 ligand (CD40l) (44) or PSGL-1 (45), CD40 and Mac-1 (integrin α_M_β_2_, CD11b/CD18) on leukocytes, markedly dampens the pathological hemostasis and inflammation including vascular inflammation and thrombosis which are key events in NEC. These findings have important implications for the development of therapeutics to treat diseases in which platelet–monocyte aggregations are implicated in the pathogenesis of NEC.

## Data Availability

The original contributions presented in the study are included in the article/Supplementary Material, further inquiries can be directed to the corresponding author.
